# Quantum biological insights into CRISPR-Cas9 sgRNA efficiency from explainable-AI driven feature engineering

**DOI:** 10.1093/nar/gkad736

**Published:** 2023-09-20

**Authors:** Jaclyn M Noshay, Tyler Walker, William G Alexander, Dawn M Klingeman, Jonathon Romero, Angelica M Walker, Erica Prates, Carrie Eckert, Stephan Irle, David Kainer, Daniel A Jacobson

**Affiliations:** Computational and Predictive Biology, Biosciences, Oak Ridge National Laboratory, Oak Ridge, TN, USA; Bredesen Center for Interdisciplinary Research and Graduate Education, University of Tennessee-Knoxville, Knoxville, TN, USA; Synthetic Biology, Biosciences,Oak Ridge National Laboratory, Oak Ridge, TN, USA; Synthetic Biology, Biosciences,Oak Ridge National Laboratory, Oak Ridge, TN, USA; Bredesen Center for Interdisciplinary Research and Graduate Education, University of Tennessee-Knoxville, Knoxville, TN, USA; Bredesen Center for Interdisciplinary Research and Graduate Education, University of Tennessee-Knoxville, Knoxville, TN, USA; Computational and Predictive Biology, Biosciences, Oak Ridge National Laboratory, Oak Ridge, TN, USA; Synthetic Biology, Biosciences,Oak Ridge National Laboratory, Oak Ridge, TN, USA; Computational Sciences and Engineering, Oak Ridge National Laboratory, Oak Ridge, TN, USA; Computational and Predictive Biology, Biosciences, Oak Ridge National Laboratory, Oak Ridge, TN, USA; Computational and Predictive Biology, Biosciences, Oak Ridge National Laboratory, Oak Ridge, TN, USA

## Abstract

CRISPR-Cas9 tools have transformed genetic manipulation capabilities in the laboratory. Empirical rules-of-thumb have been developed for only a narrow range of model organisms, and mechanistic underpinnings for sgRNA efficiency remain poorly understood. This work establishes a novel feature set and new public resource, produced with quantum chemical tensors, for interpreting and predicting sgRNA efficiency. Feature engineering for sgRNA efficiency is performed using an explainable-artificial intelligence model: iterative Random Forest (iRF). By encoding quantitative attributes of position-specific sequences for *Escherichia coli* sgRNAs, we identify important traits for sgRNA design in bacterial species. Additionally, we show that expanding positional encoding to quantum descriptors of base-pair, dimer, trimer, and tetramer sequences captures intricate interactions in local and neighboring nucleotides of the target DNA. These features highlight variation in CRISPR-Cas9 sgRNA dynamics between *E. coli* and *H. sapiens* genomes. These novel encodings of sgRNAs enhance our understanding of the elaborate quantum biological processes involved in CRISPR-Cas9 machinery.

## Graphical Abstract

**Graphical Abstract:**
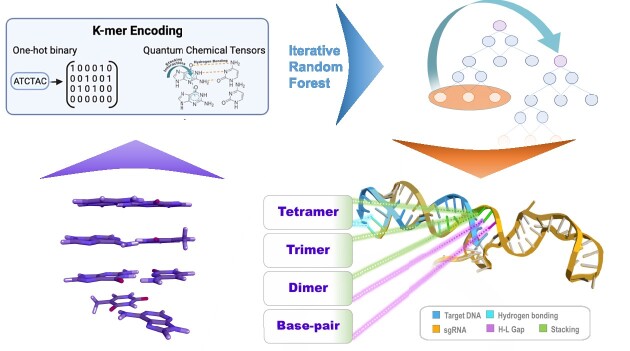

## INTRODUCTION

CRISPR-Cas9 is revolutionizing genome-editing using a single guide RNA (sgRNA) to direct precise cleavage at endogenous locations in the genome (1,2). The first step to engineer or modify a specific region using CRISPR-Cas9 is to computationally predict cutting efficiencies of potential sgRNAs. The CRISPR-Cas9 system depends on this designed sgRNA to target the protein complex to a region flanked by a 3′NGG protospacer adjacent motif (PAM). The CRISPR-Cas9 system is successful only if both specificity and efficiency occur at the target loci (3). To inform sgRNA sequence choices, genomic feature analyses have associated sgRNA attributes with cutting efficiency for CRISPR-Cas9 systems (4–8).

The efficiency of CRISPR-Cas9 systems is defined as the percentage of transgenic samples in which mutations are introduced at the intended target. Due to the time and effort required to transform many species, efficiency is critical. The calculated efficiency, while correlated, does not directly capture the specificity (on-target versus off-target cuts) that occur. Predicting sgRNA efficiency requires careful consideration of relationships among the sgRNA sequence, genomic features of the target region, and activity within the CRISPR-Cas9 system. Some of these relationships have been extensively investigated (9). Among them, nucleotide composition of the target sequence is the most thoroughly studied contributor to sgRNA efficiency (3,10–12). Specific nucleotide patterns have been associated with sgRNA efficiency; including the presence of guanine and absence of thymine near the PAM sequence, preference for cytosine near the cut site, and overall GC content (3,11,13). The seed region – defined as the five to ten bases of the target sequence nearest the PAM – is of central consideration for these patterns in sgRNA sequence composition (10,12,14).

While nucleotide sequence patterns are observed across species, their influence on CRISPR-Cas9 sgRNA association and target cleavage varies (1,15–18). The added complexities of chromatin structure have started to be considered, enhancing understandings of CRISPR-Cas9 dynamics. For example, human models were expanded with information about the insertion point within the gene sequence (19) and secondary structure of the target sequence (20,21). Target regions with low nucleosome occupancy and high chromatin accessibility have also been investigated (22–26). These structural nuances underscore even greater variation in CRISPR-Cas9 system mechanisms across organisms.

DNA is less protected in prokaryotic cells than in eukaryotic cells because of a simpler chromatin structure; and target regions are often more accessible (3). In contrast, mammalian cells have highly active non-homologous end-joining (NHEJ) systems, which induce repair mechanisms for the DNA double strand break during CRISPR-Cas9 integration. In prokaryotes, sgRNA activity is correlated with cellular survival because double stranded breaks are lethal to the cell in the absence of NHEJ (27). These pronounced differences in structure and function illuminate, in part, why models trained for mammalian species have failed to provide sgRNAs that consistently integrate with target sequences across other kingdoms. This insufficiency spurred development of organism-tailored models, including those for plants (1), yeast (18) and bacteria (28). Expanding the breadth and chemical specificity of model feature sets provides useful avenues for extending state-of-the-art sgRNA efficiency prediction to other organisms and non-model species. To achieve this next level of model prediction power, quantum chemical properties warrant consideration.

Bridging chemistry and physics, quantum chemical properties capture the ways in which electron density impact the reactivities and energetics of molecules. Some properties, such as the HOMO–LUMO gap (highest occupied molecular orbital-lowest unoccupied molecular orbital energy gap; H–L gap), describe how electron density is distributed among atoms. Meanwhile, other properties, like hydrogen-bonding energy or π-stacking energy, describe how a system's total energy changes as molecules interact. Such properties depend on how the molecular electron densities shift in response to one another. Incorporating quantum chemical detail when characterizing or predicting biological processes has been transformative for biology; providing new frameworks for viewing processes, identifying novel features, and enhancing mechanistic understandings (29,30). This work spotlights quantum properties including HOMO–LUMO gaps, hydrogen bonding, and stacking interactions to investigate the complex molecular interactions of the DNA double helix and the DNA/CRISPR-Cas9 sgRNA hybrid.

Machine learning models excel at identifying patterns in data to inform outcomes; but advances in predictive power are bottlenecked by the depth and breadth of training data. Current methods of feature evaluation for CRISPR-Cas9 efficiency are trained on experimental sgRNA cutting efficiency data from a narrow range of eukaryotic species, including human, mouse, and zebrafish (4). While these models’ species-by-species rules for sgRNA prediction are informative, their insights are rarely generalizable. Therefore, to develop advanced predictive models, the training data must be sufficiently detailed to capture the complexities of genomic structure and content that influence efficiencies of CRISPR-Cas9 integration and target cleavage.

Here we use machine learning approaches to unravel these species-dependent rules of sgRNA efficiency. Many current AI model generation approaches use techniques such as neural networks that can obscure associations behind a ‘black box’ of decision schemes. We sought to understand feature contributions to cutting efficiency for *Escherichia coli* through an explainable-artificial intelligence (XAI) approach. We used iterative Random Forest (iRF), an XAI method designed for model transparency and feature evaluation, to assess CRISPR-Cas9 efficiency and improve our understanding of the system's underlying biological mechanisms. When trained on detailed feature sets, XAI models provide a shared basis for predicting sgRNA efficiency across organisms. This work extends sgRNA efficiency modeling to assess both *E. coli* and *Homo sapiens* datasets. Additionally, our model integrates a novel and interdisciplinary feature set that includes quantum chemical properties.

## MATERIALS AND METHODS

### Datasets

#### E. coli

A publicly accessible *E. coli* dataset published by (28) was utilized. Briefly, this dataset contains 55670 unique sgRNAs that are profiled by co-expressing a genome-scale library with a pooled screening strategy. The data was compiled from three different Cas9 variations including Cas9 (*Streptococcus pyogenes*), eSpCas9, and Cas9 (△recA). The eSpCas9 is a Cas9 that has been reengineered for improved specificity and the Cas9 (△recA) was developed by knockout of recA blocking DSB repair. The dataset contains both sgRNA sequence and empirical CRISPR-Cas9 efficiency scores for each of the respective guides. The cutting efficiency scores were calculated by taking the binary logarithm (log_x_) of the selected read count to the control read count. We focused on the Cas9 dataset for analyses within this manuscript.

#### H. sapiens

A publicly accessible *H. sapiens* dataset published by (10) was utilized. This dataset contains 1278 unique sgRNAs based on a human malignant melanoma cell-line (A375) viability analysis. The cutting efficiency was determined in the same manner as described above with the log_2_ fold change calculated relative to the change in abundance during a two-week growth period. Additionally, a larger dataset curated by (33) which contains four publicly-accessible human experimental sgRNA efficiency datasets (11,19,39) including multiple cell lines (HCT116, HEK293T, HELA and HL60) was considered. Cutting efficiency values were defined as the log_2_ fold change in the measured knockout efficacy.

#### Multi-species

The multi-species model included sgRNA efficiency data from all previously described datasets. When model training occurs on datasets spanning multiple species, all data is min-max normalized on a scale of 0 to 1 and composed into a matrix of sgRNA and cutting efficiency scores for model input. To eliminate species bias due to sample size, a consistent subsampling of 15 000 sgRNAs was utilized from *E. coli* and *H. sapiens*; and the species was encoded as a binary feature.

### Feature matrix

#### Quantum chemical properties (29)

Quantum chemical properties provide unique insights into the factors influencing sgRNA efficiency in CRISPR-Cas9 systems. Canonical of DNA–DNA and DNA–RNA duplexes were modeled to assess these factors. The analysis included quantum chemical properties of individual nucleotide bases; base-pairs; and base-pair dimers, trimers, and tetramers. In this way, a fourteen-Ångstrom (4 bp) cut-off distance was invoked for long-rangeinteractions in the sgRNA. Additionally, a new sliding-window approach for the nucleotide base positions was developed for sgRNA interactions with the target DNA. Base-pair interactions were encoded into blocks, which subdivided the twenty-nucleotide sgRNA. In this approach, five ranges of interactions were assessed, from intramolecular to intermolecular.

HOMO–LUMO gap has been described as a signpost for a molecule's kinetic stability (40). It describes the energetics of allowed electron transitions, and the likelihood of processes involving electron mobility. Structurally, the H–L gap reflects a molecule's landscape of phase dependence for electronic wave function interactions—both constructive and destructive— that originate covalent molecular interactions (41). Hydrogen bonding, meanwhile, is a contextual property. It reflects an energetic preference for arrangements of molecules in relation to one another. Hydrogen bonding directs non-covalent interactions between molecules, playing roles in thermodynamic stability and the energetics of protein folding, as two examples (42). Stacking interactions are similarly contextual interactions and occur between aromatic rings. Stacking interactions range from π-π interactions within the rings—of the overlapping *p*-orbital electron density—to steric repulsions from exocyclic groups, which are implicated in DNA twisting (43). Hydrogen bonding and stacking interactions differ in the chemical species that participate (Supplementary Figure S2). Whereas hydrogen bonding interactions occur between hydrogen and a hydrogen bond acceptor, stacking interactions occur between aromatic moieties. Quantifying the energetics of these interactions complements a detailed feature set for machine learning models and sgRNA efficiency prediction.

The density-functional-based tight binding method (DFTB) is a powerful approach for large-scale atomistic simulations and calculating quantum chemical properties. This work uses the DFTB3/3ob parameter set (third order parametrization for biological and organic systems). Calculations with the DFTB3/3ob parameter set yield excellent molecular geometries (Wang and Berkelbach, 2020), which compare favorably with more resource-intensive methods. For example, DFTB3-3OB structures exhibit maximum absolute deviations of 0.045 Ångstroms from MP2/6-31G(d) methods (second order Møller–Plesset perturbation theory with six-primitive split valence polarized Pople basis; (44)).

#### Initial coordinates

Nucleotide coordinates were collected from PubChem (45). B-DNA base-pairs were extracted from crystal structure data (46) (PDBID: 167D; (46)). RNA hybrids and DNA–RNA hybrids were prepared by sterics-driven structure overlay in Biovia Discovery Studio software (Dassault Systèmes, S.E.). The nucleotide base and base-pair geometries were optimized through a gradient descent method with the simulation procedure described below. By optimizing the building blocks (the bases and base-pairs) and then systematically constructing kmers, we maintain an internal consistency of coordinates across the full (*n* = 904) set of systems. This reduction of the full conformational freedom of the kmers was motivated by the work of Šponer *et al.* (59), and serves primarily to prevent assertion of an optimal geometry where the context is not considered; and secondarily to avoid specialization to a context which may not be extensible or generalizable across the diversity of available conformational states and species-dependent DNA winding or remodeling. After optimization, each base-pair was aligned with the xy-plane in Open-Pymol software (Schrödinger, Inc.). In analogy to Gil *et al.* (47), each base-pair was then translated such that its centroid was the origin of coordinates. To complete the unambiguous set of transformations, the pyrimidine carboxyl groups provided a final constraint. For this, the thymine carbonyl bond and cytosine carbonyl carbon were rotated to be normal to one another.

#### K-mer construction

For all constructs, base-pairs were stacked at a distance of 3.5 Å along the z-axis, and rotated 36 degrees about their centroids (the origin). Structures were prepared with scripts executed in Open-Pymol. All non-chimeric single-strand combinations were assessed. In total, five nucleotide bases, size base-pairs (bp), 32 bp dimers, 156 bp trimers and 716 bp tetramers were evaluated. Compiled k-mers were assessed by single-point energy calculations using the simulation procedure described below.

#### Simulation

Calculations were carried out at the DFTB3-D3(BJ)/3ob level of theory with Grimme's D3(BJ) dispersion correction (48,49). Dispersion corrections were included to capture non-covalent interactions, resolving van der Waals and London dispersion forces in detail. Grimme's dispersion correction was selected to describe medium and short-range dispersion effects (50). Additionally, a ‘COSMO’ model was used with water as a solvent (conductor-like screening model). This model approximated solvent interactions and contextualized the geometries and energy calculations to a water environment. Total system energy, HOMO–LUMO gap, and other quantum tensors were compiled for assessment in an Iterative Random Forest model.

#### Methods justification

To our knowledge, there is no consensus method (or preferred method) for simulating oligonucleotides beyond base-pairs. For simulations with individual nucleotide bases and base-pairs, LC-DFT is indicated in preference to high-level *ab initio* methods for recovering frontier orbital energies (55). Despite its excellent performance in this context, LC-DFT typically scales as *N*^4–6^, with N the number of atoms (56,57). Furthermore, the total number of systems to sample for all canonical gRNA sequence fragments increases combinatorically with the degree of base-pair polymerization (trimers, *n* = 156; tetramers, *n* = 716). These are competing imperatives in this work which rapidly escalate the computational demand. Therefore, a low-scaling method is essential to describe even modest windows of the gRNA structure with quantum chemical detail. We select the DFTB method because it provides reliable geometries (58) with scaling (N3 (57)) that is amenable to the large number of constructs considered in this work (*n* = 904). We compare the DFTB3/3ob model output with LC-μBLYP/pVTZ (long-range corrected, range separated, Becke–Lee–Yang–Parr functional with triple zeta polarized valence basis) calculations up to base-pair dimers. We find that the top features (base-pair H-L gap, hydrogen bonding energy, base-pair stacking energy) and the regions of special interest (3′ and 5′ termini) are reproduced across models and methods (Supplementary Figure S4). Moreover, the level of model prediction is consistent (>81%) across models, with the top fifty features emphasizing quantum chemical properties (Supplementary Figure S4). We include frontier orbital and ground-state energies at DFTB3/3ob, LC-DFT/pVTZ and HF/6–31G** levels of theory (Hartree-Fock with six-primitive split valence Pople basis and d-/p-type polarization functions) to compare the ordering of features across methods (Supplemental Table 5).

#### Public resource

All calculated quantum chemical properties for nucleotides and k-mers have been compiled in Supplementary Table S1.

#### Positional encoding

Matrix generation involved extraction of several isolated feature sets. Position-independent and position-dependent encoding of the 20 bp sgRNA were performed as described by Doench et al., 2014. Briefly, position-independent features were determined by the count of nucleotides within the 20 bp sequence both as a single base (A/C/T/G) and as paired bases (AA/AC/AG/etc.). Position-dependent features were represented using binary variables (1 represents presence at that position) to encode the position of nucleotide bases up to base-pair oligomers. To describe all possible combinations, each position was described by four bits, with a binary value for each of the four possible bases (A/C/T/G). Paired bases are further encoded with a binary value for each of the 16 possible base pair combinations. Additionally, we encoded the PAM (NGG) sequence by incorporating position-independent encoding of the *N* nucleotide. The combination of positional encoding approaches resulted in 384 features for each sgRNA assessed.

Further positional encoding was conducted with k-mers to extend sequence fragment descriptions to the full guide RNA. This encoding considers combinations of fragments, capturing how the fragment's context in the full transcript influences sgRNA binding and efficiency. Here, a *k*-mer is simply a sequence of characters in a string. We utilized *k*-mers to capture nucleotide neighborhoods, considering multiple base pairs with defined dependent positions. This was done through a stepwise integration of additional nucleotides as described above in a position-dependent manner including nucleotides in groups of two to five. The binary matrix includes the positional encoding using a sliding window so that each position from 1 to 20 (less the *k*-mer length) is encoded.

#### ‘Raw’ features

Several raw value features were determined including GC content (ratio from 0–1 representing the proportion of the sgRNA sequence that is composed of GC), temperature of melting of the DNA duplex (calculated by the Watson-Crick formula of Tm(°C) = 64.9 + 41 * (nG + nC-16.4)/(nA + nT + nG + nC)), minimum free energy as a function of RNA structure (calculated with ViennaRNA; (51)), distance of the target sequence to the closest downstream PAM (utilizing the known genome assembly this was determined by the number of bases between position 20 of the sgRNA and the nearest NGG), and location relative to the target gene (represented by TSS, TTS and quartiles of gene sequence (Q1–Q4)). These calculated values resulted in an additional 5 features for each sgRNA assessed.

#### Normalization and correlation assessment

The predictive measure, cutting efficiency score, was min-max normalized to ensure transferability across models, control, methods, datasets and species. The distributions of high or low cutting efficiency scores differed between species, and these skews were maintained during normalization as to not bias the technical efficiency of one species against the other.

Additionally, values were assessed for high levels of correlation between features. When any two features resulted in a correlation higher than 0.9 one of the features was removed to eliminate the chance for split weights in feature importance during model training. The feature set utilized continuous and discrete variables with varied distributions.

### Iterative random forest model

Random forest (RF) is a non-linear regression model which incorporates an ensemble of decision trees that trace the algorithm's decision process. Iterative Random Forest (iRF) expands on the Random Forest method and is described in (52,53). Briefly, iRF is an advanced form of RF that implements a boosting and feature culling process based on the feature importance values from the previous iteration's random forest. These processes further iterate and amplify the features that repeatedly indicate high predictive capacity. The boosting process produces a similar effect to Lasso in a linear model framework. In iRF, a Random Forest is generated where features are unweighted and randomly sampled, at any given node in the decision trees, with equal probability. This process establishes feature importance scores that are used to weight features in the next forest. This iterative method provides an amplification effect, increasing the chance that important features are evaluated at any given node (52,53).

For this study, the process of weighting and generating a new Random Forest is repeated 10 times with 1000 trees and incorporates a 5-fold cross validation. For each run, the data is separated into an 80/20 training/test split where 80% of the data is used for training the model and the remaining data (not utilized in model training) is used for testing. Each feature is ranked by its importance in the tree building step and the direction of impact is determined based on the signed correlation of feature value and cutting efficiency value. Specifically, the feature matrix described above (incorporating positional encoding of the sgRNA nucleotide composition through a one-hot binary method, and quantum chemical properties) is utilized to predict the sgRNA cutting efficiency. Multiple iRF models were produced with different feature sets to assess top importance-scoring, highly-predictive features; details on these models are in Table 1.

**Table 1.** iRF model summary and metrics

Model species	Source dataset	# of sgRNAs	Feature det	# of Features	Description	Test dpecies	R2	Pearson correlation
E.coli	Guo et al. 2016	40 468 [32 374 train]	Raw	5	summary values of sgRNA sequence including GC content, Tm, MFE, gene density and distance to PAM	*E. coli*	0.0406861	0.2007612
			Onehot	5911	binary positional encoding of 20bp sgRNA nucleotide sequence	*E. coli*	0.26004285	0.4914184
			QCT	316	quantitative metrics for H-bond and HL-gap based on positional nucleotide sequence	*E. coli*	0.24183122	0.4918057
			Raw.Onehot	6916	Raw + Onehot	*E. coli*	0.26028286	0.4931724
			Raw.QCT	312	Raw + QCT	*E. coli*	0.24177446	0.4939777
			Onehot.QCT	6227	Onehot + QCT	*E. coli*	0.24905183	0.500817
			Full Matrix	6232	Raw + Onehot + QCT	*E. coli*	0.24906667	0.5019173
						*H. sapien*	0.00429969	0.06557198
			Top 5	5	Based on the full feature matrix iRF model run with E.coli data, the top feature importance scores were utilized to generate new iRF modesl with 5,10,20,40100200500 and 1000 features.	*E. coli*	0.11240746	0.3436711
			Top 10	10		*E. coli*	0.15779734	0.4019815
			Top 20	20		*E. coli*	0.2017236	0.4458406
			Top 50	50		*E. coli*	0.24529071	0.4903894
			Top 100	100		*E. coli*	0.25119027	0.4967809
			Top 200	200				
			Top 500	500				
			Top 1000	1000				
H.sapien	Doench et al. 2014	1278 [1022 train]	Full Matrix	6172	Raw + Onehot + QCT based on the H.sapien sgRNA sequence set from Doench et al. 2014	*H. sapien*	0.389120714	0.6525512
H.sapien	Chuai et al. 2018	16 749 [13 399 train]	Full Matrix	6172	Raw + Onehot + QCT based on the H.sapien sgRNA sequence set from Chuai et al. 2018	*H. sapien*	0.229489979	0.486193
H.sapien	Doench et al. 2014; Chuai et al. 2018	17 421 [13 936 train]	Full Matrix	6172	Raw + Onehot + QCT based on the H.sapien sgRNA sequence set from Doench et al. 2014 and Chuai et al. 2018	*H. sapien*	0.211671332	0.4964907
E.coli + H.sapien	Guo et al. 2016; Doench et al. 2014; Chuai et al. 2018	30000 [24000 train]	Full Matrix	6172	Raw + Onehot + QCT based on the E.coli sgRNA sequence set from Guo et al. 2016 and the H.sapien sgRNA sequence set sfrom Doench et al. 2014 and Chuai et al. 2018	*E. coli* + *H. sapien*	0.486194	0.6972761 [E.coli 0.504] [H.sapien 0.491]

#### Model assessment across quantum calculations

The iRF model was run using LC-DFT and DFTB3 quantum calculations for nucleotide base, base pair, and base-pair dimer k-mers of the sgRNA to assess the variation in model output when features were calculated at different levels of quantum theory (Supplementary Figure S5). Both the predictability of the iRF models (Supplementary Figure S5A) and the top importance features (Supplementary Figure S5B) were identified. The HL-gap, hydrogen bonding, and stacking energies are maintained as key features across the models. These properties’ locations within the 3′ region of the sgRNA are also conserved (Supplementary Figure S5B). It is observed that 94 out of the top 100 features are consistent between the two models.

### Validation

#### Oligo design

As part of another project, 120000 unique synthetic guide RNAs (sgRNAs) were synthesized by Agilent (Santa Clara, CA) as 90mers consisting of the 35-bp J23119 Anderson promoter, the 20-bp spacer, and the 35-bp 5′ end of the sgRNA. The pool of single-stranded DNA molecules received from Agilent were dissolved in 200 μl Elution Buffer (EB; 10 mM Tris, pH 8.0) and heated at 42°C until all visible solids had dissolved.

The sgRNA sequences were picked to minimize potential specificity issues. Namely, only sequences with unique seeds and no matches to sequences adjacent to non-canonical PAMs were chosen. Additionally, Cas9 was expressed at a moderate rather than a high level, which should further reduce the effects of decreased specificity.

#### Oligo processing and donor library production

Once in solution, second strand synthesis proceeded by mixing 20 μl oligo solution, 2.5 μl 10 μM oWGA139, and 25 μl NEB 2× Q5 Hot Start Master Mix into each of two 0.2 ml PCR tubes. This reaction was placed in a thermal cycler and the following program was run: 98°C 1 min, 68°C 10 s, 72°C 5 min, 4°C hold. Once the hold was reached, 2.5 μl 10 μM oWGA140 was added to each tube, and the following program was run: 98°C 1 min; 25 cycles of 98°C 5 s, 68°C for 10 s, 72°C for 15 s; 72°C for 5 min, 4°C hold. The PCR product was purified by running on a 3% agarose gel in TAE buffer until the band was halfway down the gel, removing a gel slice containing the band, and purifying the DNA with the NEB Monarch DNA Gel Extraction Kit. To clone this purified double-stranded oligo, a PCR of the pSS9-gRNA vector (62) was performed using the NEB Q5 Hot Start 2X Master Mix as manufacturer's instructions with the primers oWGA137 and oWGA138. 5 μl DpnI (NEB) was added directly to the PCR product and incubated at 37°C overnight. This reaction was cleaned by adding 45 μl water and 40 μl MagBio HighPrep PCR Cleanup magnetic beads and following manufacturer's instructions. Quality of this backbone vector fragment was empirically tested by transforming 100 ng into Lucigen E. cloni Supreme DUO electrocompetent cells per manufacturer's instructions; as ∼40 colonies were produced in this reaction, the backbone was considered suitable for Gibson assembly. 100 ng of vector backbone and 17.56 ng oligo library PCR product were added to a 20 μl NEBuilder HiFi Assembly reaction per manufacturer's instructions (a 5:1 insert:vector ratio). Afterwards, the reaction was cleaned via drop dialysis with a 0.02 micron nitrocellulose filter floated on 250 ml of 18.2 MΩ water. 2 μl of this dialysis product was added to each of 6 25-ul Lucigen E. cloni Supreme DUO comp cell aliquots, and electroporation was performed using a BioRad MicroPulser set to the *E. coli* 1 program. 950 μl Lucigen Recovery Buffer was added to each transformation, these six solutions were combined, and the totality was incubated at 37°C for 1 h. Afterwards, this recovery culture was added to 100 ml LB + 100 μg/ml carbenicillin and incubated at 37°C overnight. The next morning, the culture was harvested, the supernatant was discarded, and the cell pellets were suspended in 200 ml + 200 μg/ml carbenicillin and incubated at 37°C for four h. This fresh culture was harvested via centrifugation, then plasmid DNA was extracted using a Zymo Research Midiprep Kit. This library, referred to hereafter as the Donor Library, was sent for sequencing by Illumina.

#### Host competent cell preparation

*E. coli* K12 MG1655 containing pX2-Cas9 was struck from a freezer stock to an LB + 50 μg/ml kanamycin agar plate, and a single colony was picked to 5 ml of LB + kan in a culture tube. This culture was incubated at 37°C overnight, then it was used to inoculate 110 ml of LB + kan + 0.2% arabinose to an OD600 to 0.1. Growth was monitored over the course of 3 h, and 100 ml of the cells were harvested once an OD_600_ of ∼0.6 was measured. The cell pellet was washed 3x with 10% glycerol, then suspended to a final volume of 1000 ul.

#### Host library production

To prepare the library for transformation, 10 μg of library vector was mixed with 100 pg each of pWGA128 and pWGA130 to provide internal controls for escape and cutting, respectively. 52.5 μl of controlled library solution was added to 525 μl of electrocompetent cells, and 55 μl of this mixture was electroporated in a 1 mm gap cuvette by a BioRad Micropulser set to the *E. coli* 1 program 10 times in total, adding 950 μl of SOC to each electroporation immediately after the shock. These 10 ml of cells and media were combined into a culture tube, then 1 μl of 1 ng/μl pWGA129 plasmid was added to the culture to act as a control for washing. The recovery culture was incubated at 37°C for 1 h, then centrifuged and the supernatant aspirated. The cell pellet was suspended in then entirely transferred to 100 ml of LB + kan + 100 μg/ml carbenicillin and cultured at 37°C for 6 h. 50 ml of culture had cells harvested, washed once with 50 ml DNAse I Wash Buffer (10 mM Tris, 2.5 mM MgCl_2_, 0.5 mM CaCl_2_), then the pellet was suspended in 1.5 ml of DNAse I Wash Buffer and transferred to a 1.5 ml microcentrifuge tube. This pellet was washed two more times with 1.5 ml DNAse I Wash Buffer, then suspended in 990 μl of the same. 10 μl of NEB DNAse I was added, and the reaction was incubated for 15 minutes at 37°C. The cell pellet was harvested, and plasmid DNA was extracted using the NEB Monarch Miniprep Kit. This library, referred to hereafter as the Host Library, was sent for sequencing by Illumina.

#### Illumina library preparation

For both Donor and Host Library plasmid pools, PCR using ‘phased primers’ amplified the gRNA spacers to be sequenced. Briefly, five forward and five reverse primers were made, each that bound to the same site of the library plasmids but possessing zero to four additional random bases at the 5′ end; the purpose of these extra bases is to alleviate issues inherent to sequencing amplicons on Illumina platforms, as the very low complexity of amplicon molecules prevents high-quality base calls from being made by the Illumina system. These forward and reverse primer mixes were used together with 30 ng of library vector in a Q5 Hot Start PCR as per manufacturer's instructions. The PCR product was purified on 3% gel and isolated using the NEB Monarch DNA Gel Extraction Kit, quantified using the Promega Quantus fluorometer and the QuantiFluor ONE dsDNA System, and then used in the NEBNext Ultra II DNA Library Prep Kit for Illumina. During the amplification step, one forward and one reverse primer per library from the NEBNext Multiplex Oligos for Illumina (Dual Index Primers Set 1) kit was used to complete the protocol.

#### Illumina library sequencing

Sequencing libraries were validated on an Agilent Bioanalyzer using a DNA7500 chip, and the final library concentration was determined by Qubit (Thermo Fisher Waltham, MA) with the broad range double stranded DNA assay. A paired end sequencing run (2 × 75 bp) was completed on an Illumina MiSeq instrument (Illumina, San Diego, CA) with 20% PhiX spike in using v3 chemistry following the manufactures denaturing and loading recommendations. The Donor Library was sequenced using three separate sequencing runs, while the Host Library was sequenced using two separate runs.

#### Data processing

Paired end reads were merged using the USEARCH *-fastq_mergepairs* command with the *-fastq_maxdiffs* option set to 10 (63,64). The merged pairs were then matched to the original design file in FASTA format using USEARCH (*usearch -usearch_local path/to/reads.fastq -db path/to/designs.fasta -strand both -threads 30 -id 0.9 -top_hit_only -target_cov 1 -userout path/to/output.tsv -userfields query + target + id + qrowdots + trowdots + qrow + trow*). The output .tsv file was parsed in Python to count the instances of perfect reads for each design in the design file and to calculate the abundances of perfect reads for each design (calculated as number of perfect reads for a design divided by the number of merged pairs used in the initial USEARCH matching, and these data for all runs were combined into a single spreadsheet. In that spreadsheet, new columns to calculate values for the average donor library reads, average donor library abundance, average host library abundance, host wash control correction, delta, log_2_delta, and P-value for each design. Briefly: average donor library reads, donor abundances, and host abundances were calculated with the AVERAGE function in Excel; the host wash control corrected abundance was calculated as the average host library abundance minus the product of the ratio of the average host abundance of the pWGA129 wash control sequence, the ratio of the total mass of DNA used (1 μg) to the ratio of wash control plasmid added to the recovery (1 ng), and the donor abundance of the cassette; delta was calculated as the host wash control corrected abundance divided by average donor library abundance; log_2_delta was calculated by log2 delta; and the *P*-value was calculated by the Student's *t*-test function in Excel for a two-tailed heteroscedastic test. Designs possessing either less than 10 average donor library reads or a *P*-value equal to or greater than 0.05 were moved to ‘graveyard’ tabs in the spreadsheet and excluded from any further analysis. The resulting validation data are contained in Supplementary Table 6.

The iRF model was run using the same criteria as described in the ‘Iterative Random Forest Model’ section above. A classification iRF model using five-fold cross-validation with 1000 decision trees per random forest and ten iterations was trained with the Guo *et al.* Cas9 sgRNA dataset. Classes were defined by sgRNAs with bad (0) or good (1) cutting efficiency scores as determined by the 2500 guides at each tail of the Log2 normalized score distribution. The cross-validation test resulted in an ROC AUC of 0.8606. This model was then used to predict the class for a novel 120k sgRNA *E. coli* dataset as described above. Specifically, the 2500 guides at either tail of the Log_2_ normalized score distribution were assessed and found to have an accuracy of AUC = 0.7633. See supplemental for classification model test results (Supplementary Figure S5).

### Advanced feature engineering metrics

In combination with iterative Random Forest, in-house scripts for advanced interpretation of machine learning output were utilized:

*Random intersection trees (RIT)* uses binary predictor variables to identify interactions among features in a model. In short, RIT starts by collecting the full set of matrix variables in a ‘high level interaction’. Classes of observations are assigned to each variable and then, through iteratively making a random observation and identifying which variable were predictive of that observation, the interaction set is narrowed to a subset of patterns in the prediction variables (53,54). This process is repeated for each observation class. The algorithm works by assessing the node-split forest paths from iRF to find features that occur consecutively along the path (more often than would be expected by chance). The result of RIT is a set of interactions that are retained with high probability, potentially in a non-linear manner, and are therefore considered informative to the model as a whole when the features are in combination.

For these analyses, we use internal R and Python scripts designed for iterative and extensive tree-based random forest decision processes. These scripts extend conventional RIT methods for enhanced feature engineering and model comparison. They provide metrics for feature interpretation, including normalized importance scores (for comparison across models), and feature effect scores (the magnitude and direction of that feature's influence on the model). We also identify and characterize interacting features. For this, the number of samples captured, RIT (prevalence of a feature set in the model), adjusted RIT (the difference between the model and expected prevalence of a feature set), and set importance, are key indicators.

## RESULTS

### Feature importance with iterative random forest

We assess iRF-based predictive models that leverage quantum descriptors for multiple degrees of base-pair polymerization. This data captures interactions within the sgRNA nucleotide sequence, along with properties of the individual bases. This approach combines the increased interpretability of XAI methods for feature interpretation with the novel incorporation of quantum chemical properties to further mechanistic understandings of CRISPR-Cas9. Models were generated for sgRNA efficiency in *E. coli* and *H. sapiens*. Variations in feature importance across kingdoms were also assessed.

**Figure 1.**
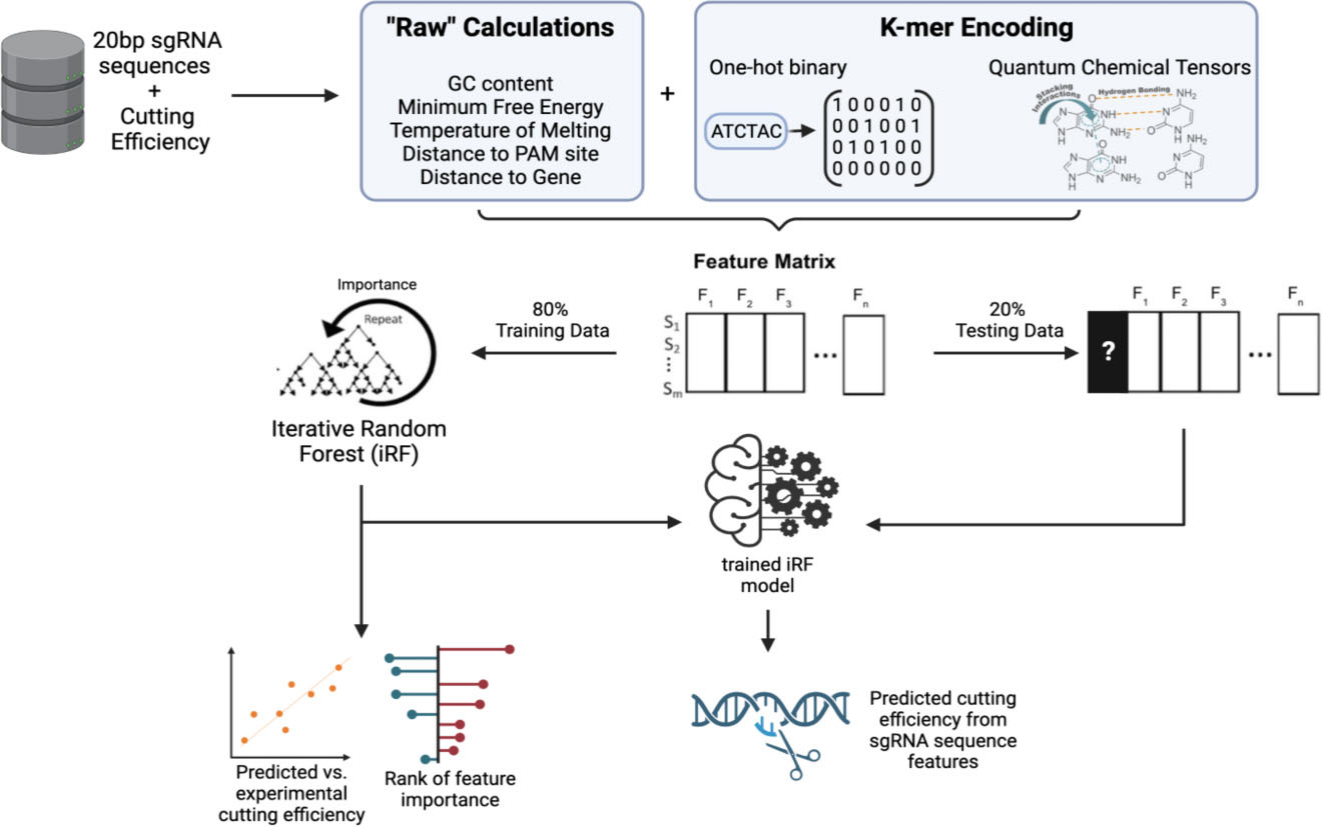
Explainable-AI method for analysis of feature importance on prediction of sgRNA efficiency. Features are formatted to generate a wide matrix with rows representing each sgRNA, corresponding experimental cutting efficiency scores and columns for all feature values. This information matrix is analyzed with an iterative Random Forest (iRF) method.

A publicly available *E. coli* dataset was used to generate a detailed feature matrix to determine highly influential properties for predicting CRISPR-Cas9 cutting efficiencies (Figure 1). In this model, the dependent variable (Y-vector) is the experimental cutting efficiency score for each sgRNA. We used quantum chemical features of nucleotides to capture the intricacies of the multi-step CRISPR-Cas9 mechanism. In addition, base-pair oligomers (*k*-mers) up to tetramers were incorporated, describing nucleotide position in the sgRNA structure with binary encoding (one-hot k-mers) and quantum chemical properties (quantum *k*-mers). These k-mers assess the subsequence structure of the 20bp sgRNA by segmenting the sequence into fragments of one to four bases. These features provide a basis for interpreting the influences of independent and dependent nucleotides on sgRNA efficiency, and progress structurally informed understandings of sgRNA efficiency. Features previously determined to influence CRISPR-Cas9 efficiency in mammalian species were also encoded, including GC content, melting temperature, and one-hot encoding of single and paired nucleotides in the target sequence. We also included metrics such as the distance between the target sequence and the nearest PAM (NGG sequence) and location of the target sequence relative to the nearest gene. To sharpen insight into the contributions of each feature, predictive capabilities were assessed by Pearson correlations and accuracy (*R*^2^) metrics (Supplemental Table 1). The iRF model using the complete feature matrix results in a predictive accuracy of 0.51, which is competitive with the most predictive models currently available. Quantum k-mers and one-hot k-mers contribute the largest feature set contributions, isolating essential features for sgRNA engineering.

### Feature engineering highlights the role of quantum mechanics

**Figure 2.**
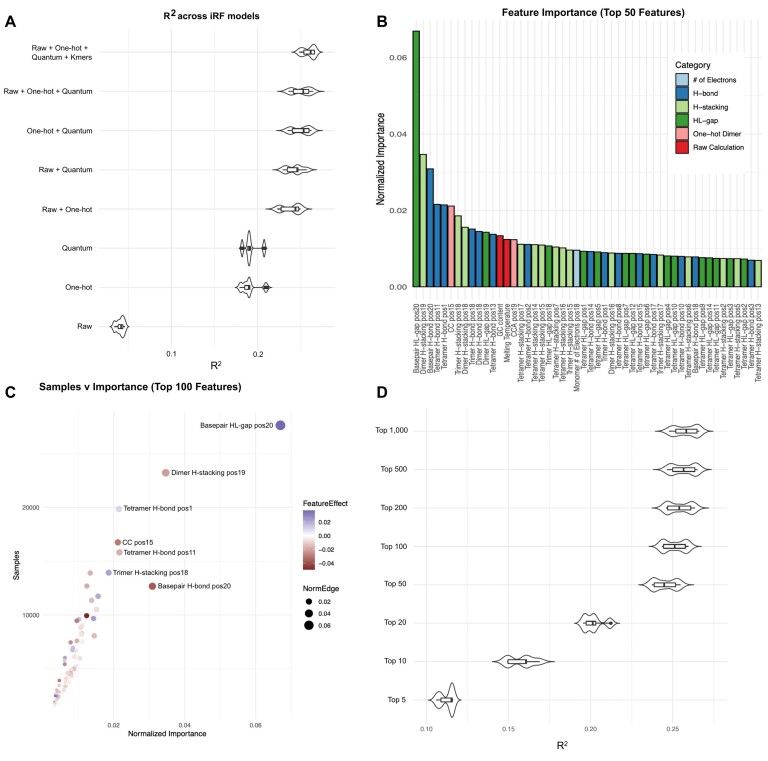
Identifying model variation based on feature input and assessing feature importance in *E. coli*. (**A**) Violin plot of *R*^2^ values based on iRF model generation with isolated feature input (feature categories described in Table 1). (**B**) The top 50 features from the full feature matrix iRF model ranked by normalized feature importance score and color-coded by feature category. (**C**) Dot plot of features from full feature matrix iRF model showing the number of samples (sgRNAs) that were influenced by that feature (y-axis) versus the normalized importance of the feature (x-axis). Color temperature increases with the feature effect score (red, negative; blue, positive) and dot size is scaled by the normalized importance score. (**D**) Violin plot of *R*^2^ values for the top 5, 10, 20, 50, 100, 200, 500 and 1000 features, based on full feature iRF model output. There is a plateau of information gained from including features with low importance scores.

A feature engineering approach clarifies the factors influencing sgRNA efficiency by identifying the model's most important variables. A total of 6232 features were used in an iRF model for *E. coli* (the full *E. coli* feature set; Supplementary Table S2). This model was trained on 32 374 sgRNA and tested on 8094 sgRNA sequences. This complete feature matrix cast as an iRF framework resulted in significant correlations between predicted and experimental sgRNA cutting efficiency values (Figure 2A). Furthermore, high prediction levels were found in the iRF model using only the quantum chemical properties feature set; and accuracy increased incrementally as additional features and k-mers were incorporated (Figure 2A; Supplementary Figure S1; Supplemental results). Below, we focus on a subset of features that were the most predictive of sgRNA efficiency (Figure 2B, C).

Based on feature importance values produced by iRF (Supplementary Table S4), top features emphasized positionally-encoded k-mers of quantum chemical properties and one-hot encoding of the target sequence (Figure 2B). These top features were maintained as highly important across two quantum chemical methods (Supplementary Figure S5). Top features were localized to positions 18 through 20 of the target sequence. This region is proximal to the sgRNA tailpin structure, the target DNA PAM sequence, and cut site for the Cas9 nuclease. The most important feature was the HOMO–LUMO energy gap for the base pair at position 20 of the target sequence (Figure 2B). This feature alone accounted for more than 6% of the variance in empirical sgRNA efficiency. The next most important feature was the base-pair dimer stacking energy at bases 19 and 20; accounting for ∼3% of the variance. Hydrogen bonding energy of the base pair at position 20 shows a similar contribution. Following these features in importance, we observe several position-dependent base pair dimer, trimer, and tetramer quantum chemical properties. Each of these features accounted for 1–2% of the dataset variance. Several one-hot encoding sequences were also important features, including cytosine positions 15 and 16 (CC pos15) and a CCA beginning at position 19. Additionally, several features with high feature importance scores are consistent with trends in the literature, including GC content and melting temperature (31).

Each decision tree within an iRF model selects features based on their contribution to the predictive ability against the experimental dataset. Because of complex relationships between features and sgRNA, individual features may not be influential for each sgRNA in the model. Therefore, each feature's average number of affected sgRNAs was also calculated for all decision trees within the iRF model. This average was compared with the total number of training data sgRNAs to determine the relative proportion of sgRNAs that each feature influenced (Figure 2C). The twenty highest-magnitude features affected between 20% and 85% of sgRNA samples. The three most-commonly influential features included the base pair 20 HOMO–LUMO gap energy, the base-pair 19–20 dimer hydrogen bond energy, and the base-pair 1–4 tetramer hydrogen bond energy (Figure 2C). These top features span both positive and negative associations with predicted cutting efficiency scores.

### Assessing feature association with sgRNA efficiency

**Figure 3.**
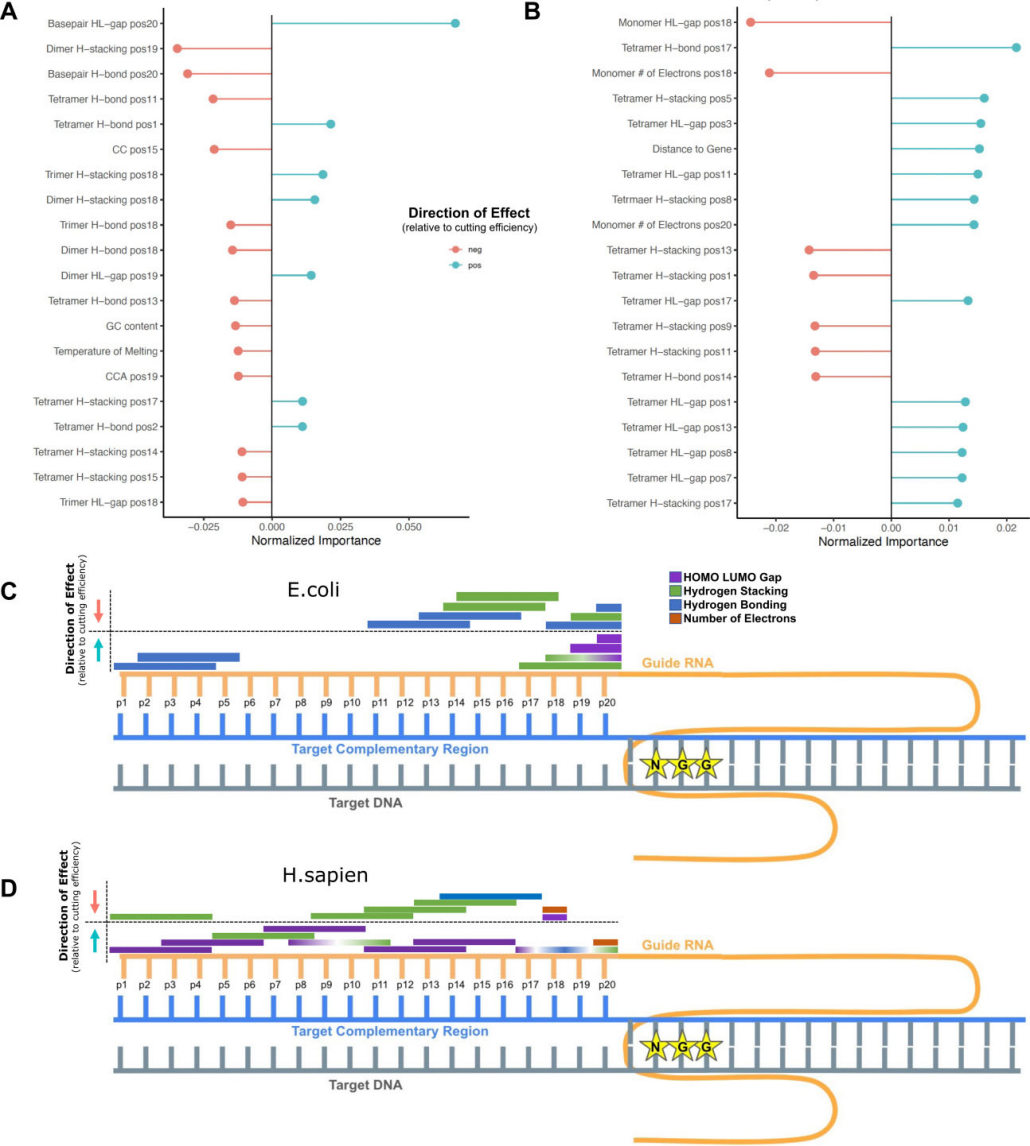
Explainable-AI interpretation through iRF output metrics and features’ directional influence on cutting efficiency. (A, B) The top 20 features from the full feature matrices ranked by normalized importance score and color-coded by the direction of the effect. Positive correlations with the cutting efficiency score are blue while anti-correlations with cutting efficiency score are pink for *E. coli* (**A**) and *H. sapiens* (**B**). (C, D) sgRNA-DNA interaction highlighting quantum chemical features of top importance, their locations, and correlated associations with cutting efficiency scores in *E. coli* (**C**) and *H. sapiens* (**D**). DNA strand represented in gray (target sequence) and blue (target complementary sequence), sgRNA shown in yellow, and PAM sequence displayed with NGG stars. The feature effect direction is indicated with arrows, up (blue arrow) indicates a positively correlated relationship between the feature value and the cutting efficiency value. Feature bars indicate quantum properties (HL gap, purple; Stacking interactions, green; H-bonding, blue) and the length of the bar indicates the *k*-mer size. Multi-colored bars indicate the same k-mer at the same position has multiple features assessed as highly important. The *E. coli* (C) model shows extensive localization of important features, primarily bp, trimer and tetramers at positions 11–20. Hydrogen bonding has outlier importance at position 1–5. Hydrogen bonding and stacking energy features are observed in both correlated and anti-correlated relationships with cutting efficiency (depending on their *k*-mer and position) while HL-gap is consistently a positive relationship nearest the PAM sequence. The *H. sapiens* (D) model has lesser feature localization, with many features overlapping in positions 5–15. For features of high importance (hydrogen bonding, stacking energy, and HL-gap), the feature-specific directional effects span both positive and negative relationships with cutting efficiency, dependent on the feature length and position. Similarly to the *E. coli* model, bp, trimers and tetramers are the most predictive. The number of electrons *H. sapiens* a top feature for the *H. sapiens* model that is not among the top feature in *E. coli*.

Each feature was assigned a direction (positive or negative) and effect size, calculated with a random intersection tree (RIT)-based approach (32) (Figure 3A, Supplementary Figure S3). These components describe a feature's relationship with the cutting efficiency score, allowing for greater interpretation of that feature's role in the CRISPR-Cas9 mechanism. For example, it has been shown that higher melting temperatures and greater GC content decrease guide efficiency (31). This anti-correlated relationship is demonstrated in our model by a negative feature effect value (Figure 3A, C). Among the important features, both positive and negative correlations with the predicted cutting efficiency score were observed (Figure 3).

The top positional encoding features also showed varied directions of correlation with sgRNA cutting efficiency. Two essential features are the HOMO–LUMO gap and the hydrogen bond strength at position 20 between the sgRNA and target sequence (Figure 3A, C, Supplementary Figure S2). The HOMO–LUMO gap is positively correlated with sgRNA cutting efficiency, while the hydrogen bond strength at the same position is anti-correlated. Further, the direction of the hydrogen bond strength feature effect value varies with position and encoding length—whether base-pair monomers, dimers, trimers, or tetramers are considered. Hydrogen bond strength at positions 18–20 have negative effects, while hydrogen bond strength at position 1 has a positive effect. This contrast indicates varied preferences for hydrogen bonding energy across regions of the target sequence. One-hot encoding indicates position 15 CC as anti-correlated, while position 19 GC is positively correlated with sgRNA cutting efficiency. Additionally, our model indicates that increased distance to PAM is anti-correlated with sgRNA cutting efficiency.

### Quantum chemical insights into kingdom-specific dynamics

Current species-trained models are inadequate for prediction across organisms. To assess the organism specificity of our iRF model, we tested the efficacy of the full *E. coli*-trained model on several publicly accessible *H. sapiens* datasets (10,33). The resulting predictions were extremely poor, with a Pearson correlation of 0.016. This supports that key features identified by models trained on experimental data from a single species are not predictive across species, particularly where varied CRISPR-Cas9 interactions and complex DNA structures contribute.

*E. coli* and *H. sapiens* represent different kingdoms, Eubacteria and Animalia. These classifications span single-celled to multi-celled organisms; varied organellar makeup; and diversity in genomic and epigenomic structures and compositions. To compare the predictive capability of the newly integrated feature set across kingdoms, we generated a model trained on *H. sapiens*-specific data (10,33). The full feature matrix was generated as described for the *E. coli* model, using the specified sgRNA sequences in the Doench et al. 2014 (1278 sgRNAs) and Chuai et al. 2018 (16749 sgRNAs) datasets (Supplementary Table S3). The iRF model was prepared with the same five-fold cross validation scheme as for the *E. coli* models. The resulting model had a Pearson correlation of 0.50 (Table 1). This is competitive with several of the top predictive cutting efficiency models currently available for human genome editing (4).

To explore this result, we cross-examined the twenty highest-scoring features for each species-trained model (Figure 3). Similarly to those identified in the *E. coli* model, quantum chemical tensors in the target sequence's seed region (sgRNA position 10–20, closest to the PAM sequence) appear to drive the *H. sapiens* model prediction (Figure 3B). While quantum chemical properties as a feature set are important for sgRNA efficiency in both *E. coli* and *H. sapiens*, the most predictive features vary considerably between models. In *E. coli*, the occupancy of frontier orbitals for PAM-adjacent nucleotides was a driving factor in CRISPR-Cas9 cutting (Figure 3C). In the *H. sapiens* model, however, properties in central regions of the target sequence were highlighted (positions 5–15; Figure 3D). Key features for this model emphasize hydrogen bonding energy and stacking interactions, along with electron occupancy (Figure 3B/D). These features signpost novel mechanistic interpretations focused on central regions of the target sequence that can be explored in future biological studies.

## DISCUSSION

Current sgRNA efficiency prediction models are limited by a narrow range of species data for training. To enhance the predictive accuracy, many models use deep learning techniques that can obscure interpretability of their feature effects. This work expands understanding of the factors influencing sgRNA efficiency for a bacterial dataset with an explainable-AI method. Furthermore, we incorporate quantum chemical properties to provide novel insights into sgRNA efficiency, dynamics, and propose interpretations for the CRISPR-Cas9 mechanism.

A panel of *E.coli* sgRNA sequences were encoded into a matrix incorporating PAM sequence distance, sgRNA melting temperature, GC content, one-hot binary encoding, and quantum chemical properties. This detailed feature set was used to train an XAI iRF model against experimentally calculated cutting efficiencies. The goal of this model was to predict on-target CRISPR-Cas9 activity to better understand sgRNA efficiency in the genome. The predictive capacity of the machine learning model was enhanced by advanced k-mer features (binary and quantum) and is competitive with currently available models. The XAI methodology permitted investigation of the underlying features by quantifying feature importance scores.

Quantum chemical properties carried the highest importance for prediction of sgRNA efficiency. This feature set is novel in the domain of CRISPR-Cas9 models and enhances the model's biological interpretability. Beyond position-specific sequence information, which is commonly encoded in a binary matrix, quantum chemical properties signpost the varied nucleotide interactions that mediate the CRISPR-Cas9 mechanism. The sgRNA seed region featured quantum properties with high predictive capacities. Descriptors of hydrogen bonding energy, stacking interactions, and HOMO–LUMO gaps enrich the interpretation of why this region plays a vital role in CRISPR-Cas9 efficiency. Particularly, we note indications of mechanistic competition for preferred structural features. We focus on three main themes: locality in the ‘seed region’, degree of base-pair polymerization, and mechanistic competition (Figure 3C).

The ‘seed’ region, the five to ten base pairs on the target sequence's 3′ end nearest to the PAM sequence and cleavage site, has been a focus of sgRNA construction across mammalian species. Several of the top *E. coli*-based features, especially quantum properties for base-pair and dimer structures, are essential in positions 18–20 (Figure 3A; Figure 3C). In contrast, tetramer features in the seed region are highlighted further from the PAM sequence. These differences suggest a structure-activity relationship and may indicate separate mechanistic steps involving these regions.

Looking to the multi-step CRISPR-Cas9 mechanism, we postulate that considering both DNA-DNA double helix unwinding and subsequent DNA–RNA binding are essential for interpreting these results.

This distinction can be seen when interpreting a positive correlation between the hydrogen bond stacking energy at position 18 of the target sequence (Figure 3A). Mechanistically, this indicates that position 18 is important for DNA–RNA binding. Once helix melting has been initiated at the target sequence's 3′ end, the remaining sequence composition is potentially less important for unwinding. Therefore, we suggest that while lower hydrogen bond strength at position 20 is energetically preferable for DNA double helix melting, higher hydrogen bond strength at position 18 is important for strong DNA–RNA binding (Figure 3A).

In another view, a positive correlation between the HOMO–LUMO gap energy and cutting efficiency is observed at position 20. The HOMO–LUMO gap may capture conformational changes that are occurring during the initial integration of the CRISPR-Cas9 complex. We note recent work identifying the ‘phosphate lock loop’ in this interpretation. When the PAM sequence is identified and bound, the DNA kinks to enable DNA helix unwinding and permit DNA–RNA binding. These structural events are stabilized by a phosphate lock loop proximal to the PAM (34–37). While a high HOMO–LUMO gap in this region may describe a change in molecular stability, the weaker hydrogen bonding may relate to the DNA double helix unwinding that follows.

A further discovery was the variation in influential properties for sgRNA efficiency across species. The novel quantum chemical property feature set is transferable across species because of its construction from simple nucleotide sequences. While the model does not provide a comprehensive view of nucleotide binding and interactions in complex genomes, it does provide a structural grounding for mechanistic interpretations that cannot be captured with conventional binary encodings. Species-tailored iRF models generated utilizing *E. coli* and *H. sapiens* data exemplify this increase in interpretability. Moreover, the *H. sapiens* model provides sufficient predictive power while also allowing for feature engineering insight. Such analytical interpretability is not currently available with other top predictive models in the field due to their deep learning focus (38). Furthermore, our model performance points to the beneficial integration of quantum chemical properties, not only for interpretation but also for sgRNA efficiency prediction.

This novel feature set was shown to be of high importance across species; with each species model emphasizing different quantum chemical properties or locations of interest. This highlights uses for understanding complex mechanisms across diverse species and datasets. For example, recent work, including that by Palermo *et al.*, has made strides in its combination QM/MM description of the *S. pyogenes* CRISPR-Cas9 system, with particular focus on the scissile phosphodiester bond [1, 2]. In light of our work, sampling a larger region of the guide and a range of transcript candidates, focusing on the *p*-20 to *p*-15 region, could provide powerful insight into the *S. pyogenes* CRISPR-Cas9 mechanism and its relationship to guide sequence composition (60,61).

Variation in feature importance across models supports critiques that organism-tailored models are not transferable across species. This was further emphasized by the very low performance of the *E. coli* trained model as a predictor of *H. sapiens* sgRNA efficiency. Not only is model exploration across species needed; new datasets with standardized scoring methodologies are required for such studies to be conducted. Current publicly accessible cutting libraries vary across factors that can greatly influence the calculated cutting efficiency scores; including sgRNA sequence length, type of assay, and the use of a dead Cas9 (dCas9), Cas9 with a non-complementary sequence as the guide, no Cas9 or no guide as a control. Each of these variations influence the reported efficiency values and complicate synthesis of multiple datasets within a single model or comparison across datasets. There are further challenges in assessing cutting specificity in bacteria due to the general lack of NHEJ machinery. This causes many double stranded breaks to result in cell lethality. Future work will generate genome-wide cutting efficiency libraries to expand on currently available data as well as standardize libraries across species for comparison and model integration.

This work established a novel feature set, with quantum chemical tensors, that advances the mechanistic interpretation and predictive accuracy of the model and will become a resource for continued work in the field. Initial insights into essential variables for understanding the CRISPR-Cas9 mechanism have been identified through feature engineering techniques. These advances provide avenues for improving CRISPR-Cas9 sgRNA generation and identify points of interest for experimental follow-up in the CRISPR-Cas9 mechanism. Furthermore, these advances provide insights into species variation of CRISPR-Cas9, and indicate methods for predictive model enhancement.

## Supplementary Material

gkad736_Supplemental_files

## Data Availability

No new data were generated or analysed in support of this research, except for guide RNA sequences and depletion scores, which are included in supplemental material. Source code for data processing can be found on GitHub under an MIT license. Code for generating the feature matrix can be found at https://github.com/nosha003/sgRNA_iRF. Code for iterative Random Forest (iRF) can be found at https://github.com/jailGroup/RangerBasediRF.

## NOTES ADDED IN PROOF

This manuscript has been authored by UT-Battelle, LLC under Contract No. DE-AC05-00OR22725 with the U.S. Department of Energy. The United States Government retains and the publisher, by accepting the article for publication, acknowledges that the United States Government retains a non-exclusive, paid-up, irrevocable, world-wide license to publish or reproduce the published form of this manuscript, or allow others to do so, for United States Government purposes. The Department of Energy will provide public access to these results of federally sponsored research in accordance with the DOE Public Access Plan (http://energy.gov/downloads/doe-public-access-plan).
