# Supplementary material for: Quantum biological insights into CRISPR-Cas9 sgRNA efficiency from explainable-AI driven feature engineering
Source: Nucleic Acids Res. 2023 Sep 20;51(19):10147–61. doi: 10.1093/nar/gkad736 (PMC10602897; doi:10.1093/nar/gkad736)
Supplement: gkad736_Supplemental_files [file gkad736_supplemental_files.zip › Supplemental_figures_and_legends.pdf]

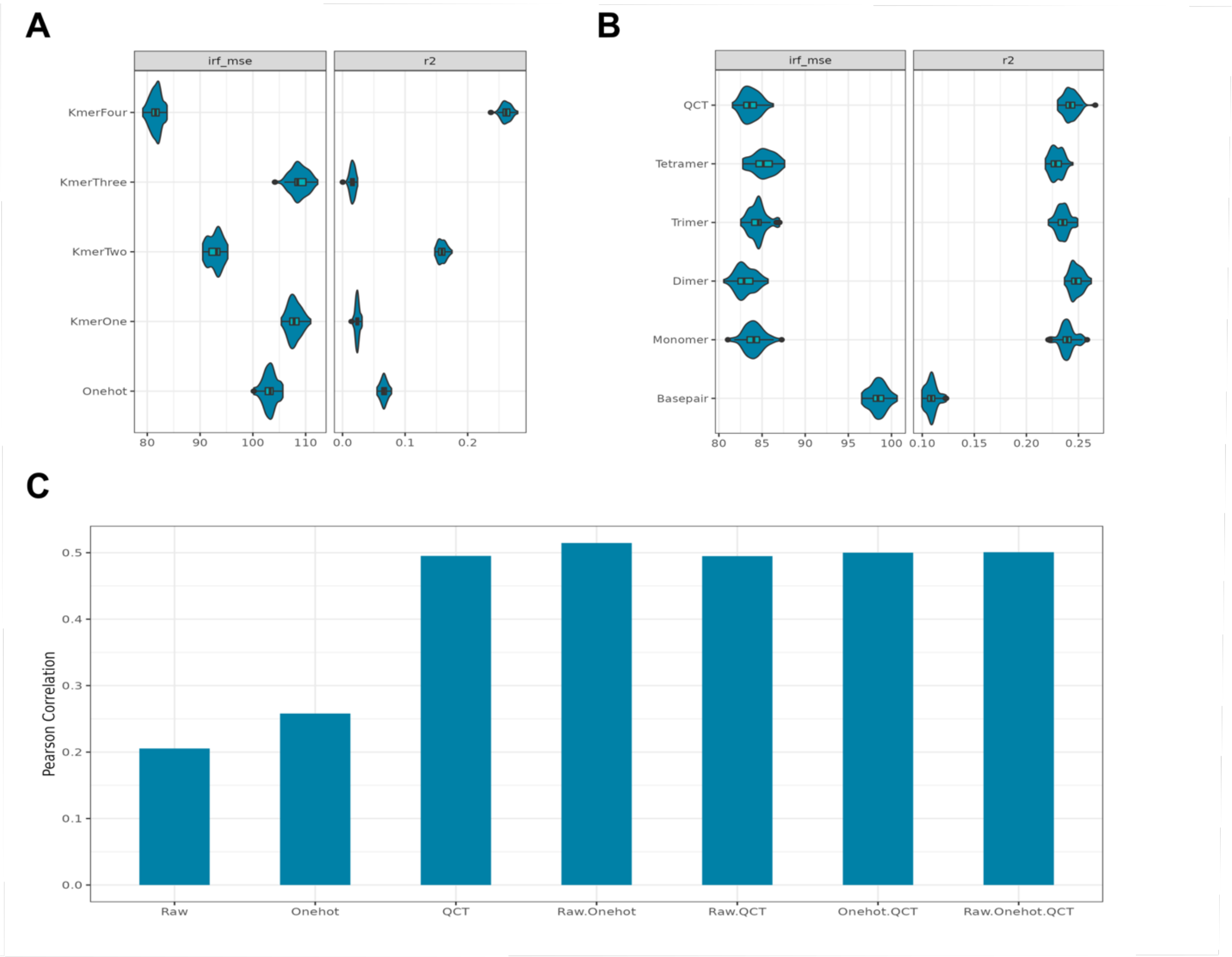

Figure S1: iRF metric output relative to feature set. Model output metrics including R squared and mean squared error (MSE) of the predicted versus experimental cutting efficiency scores for A) k-mer integration of positional encoding features and B) k-mer integration of quantum property features. C) Bar plot of the Pearson correlation predictive metric from iRF output based on the feature matrix utilized and the corresponding number of features incorporated in that matrix (line plot) showing that the increase in matrix size is not the main contributor to high predictive metrics.

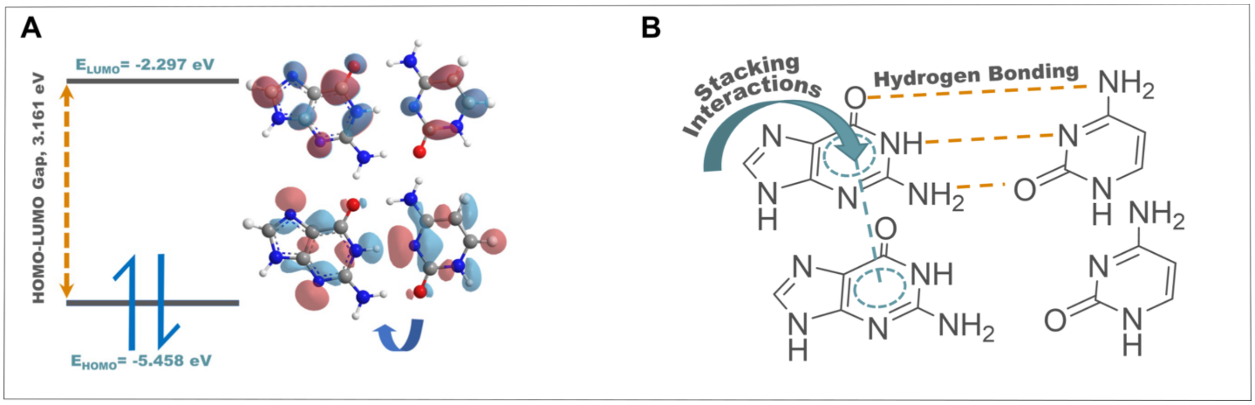

Figure S2: A) The identification of the highest and lowest occupied molecular orbitals for quantum property calculations (left). A visual depiction of the HOMO-LUMO gap based on the nucleotide organization of the sgRNA sequence (right). B) Schematic depiction of hydrogen bonding property and stacking interactions.

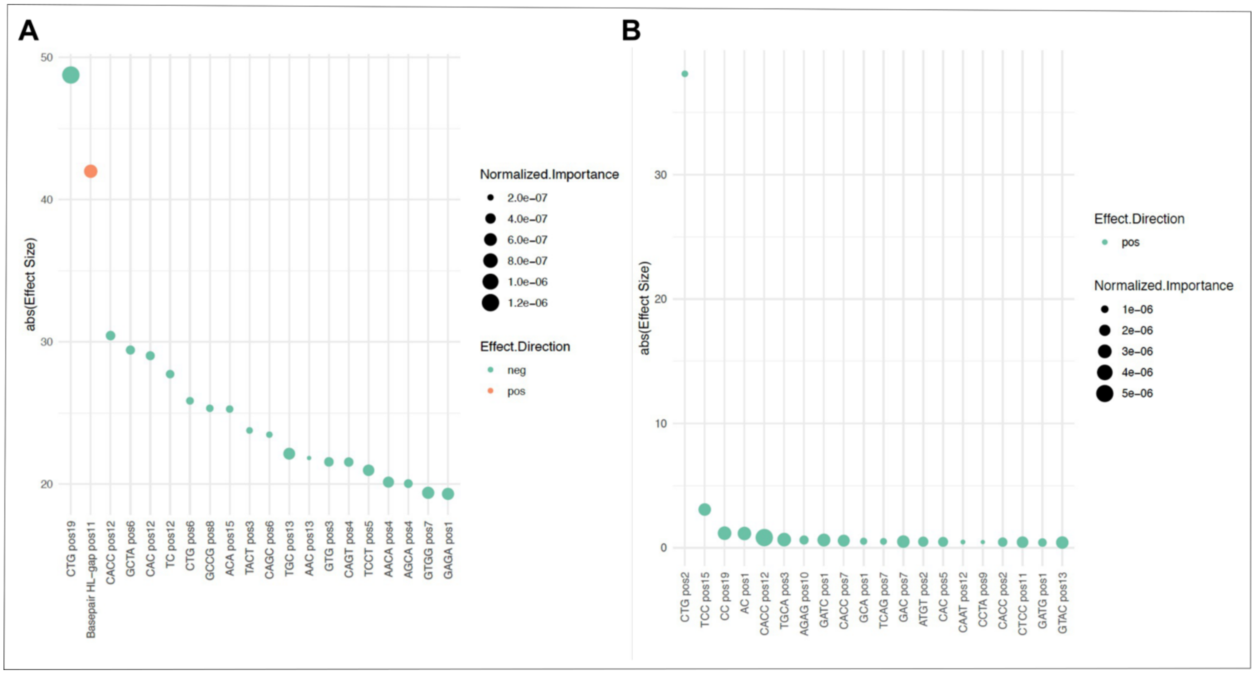

Figure S3: Effect size and direction for A) *E. coli* and B) *H. sapien*. Dot plots of top 20 features based on effect size, calculated as the average proportion of sgRNAs that were influenced by that feature based on the presence in decision trees of the iRF model. Additional information displayed includes the direction of effect (positive = orange, negative = green) and importance score (size of dot).

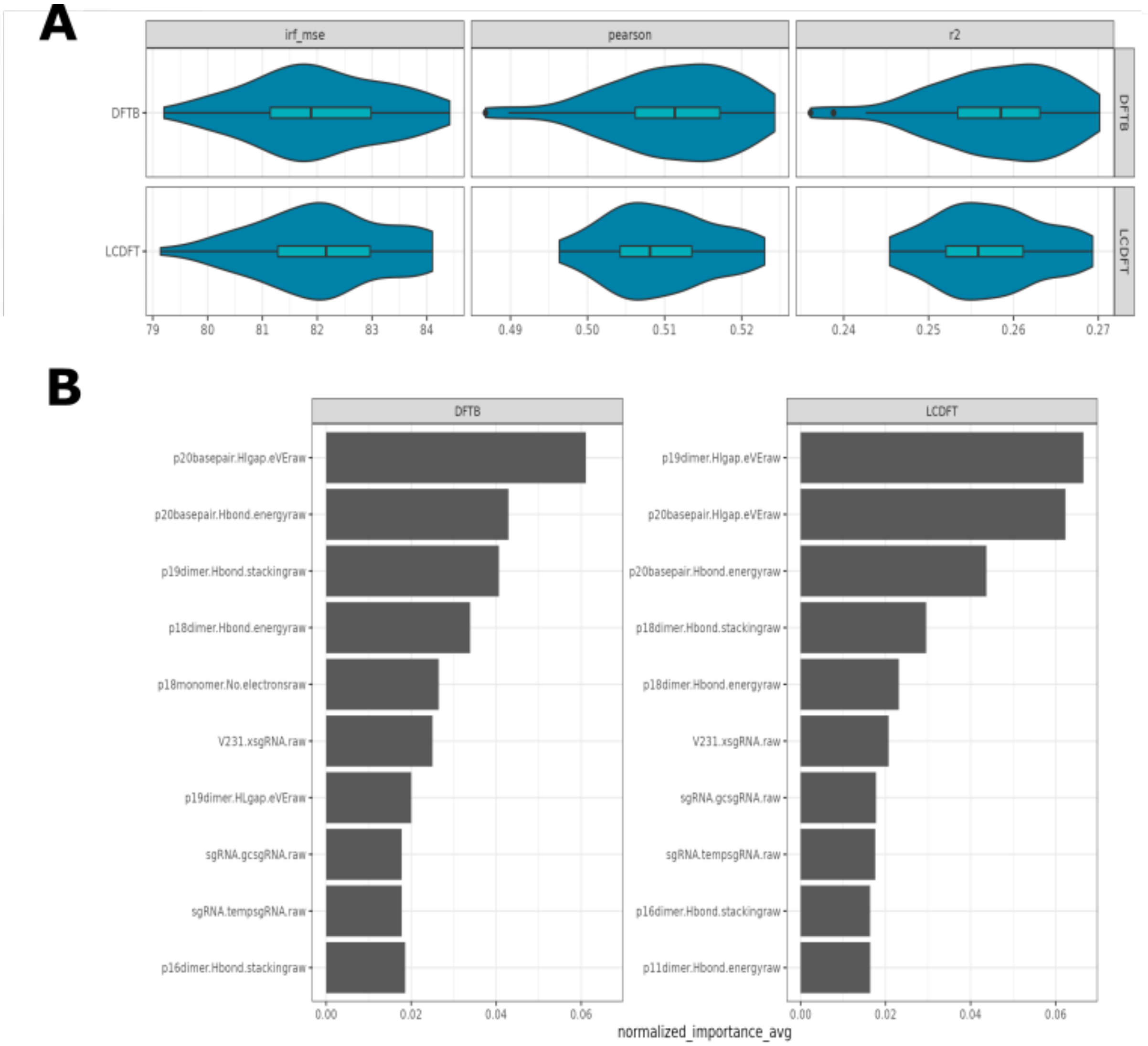

Figure S4: iRF quantum validation of DFTB3 versus LC-DFT calculations. A) Model output metrics including mean squared error (MSE), pearson correlation, and R squared of the predicted versus experimental cutting efficiency scores when iRF was run utilizing the DFTB3 and LC-DFT levels of theory for quantum calculations. B) The top 20 features from the iRF model, ranked by normalized importance score, when run with DFTB3 (left) and LC-DFT (right).

A
B

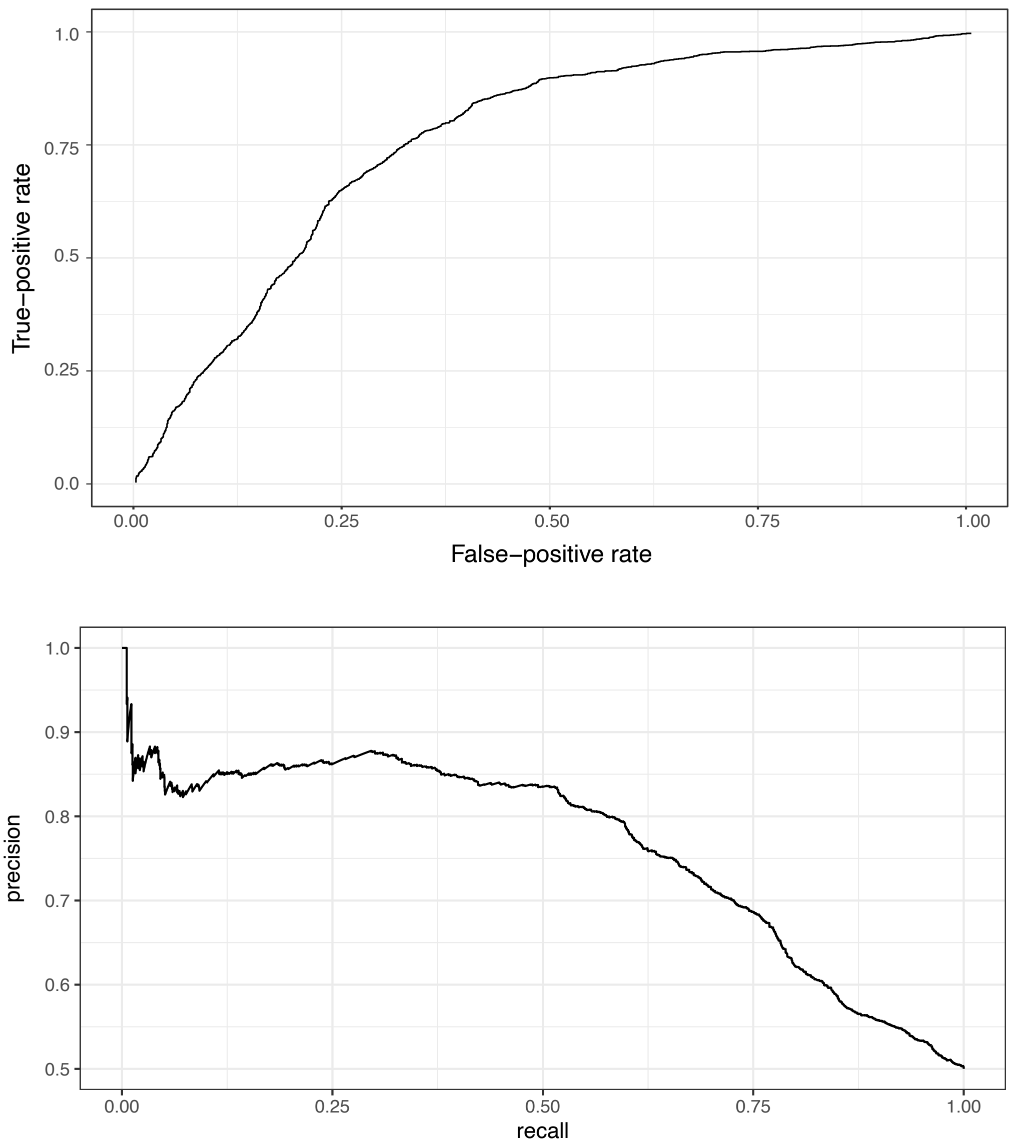

<u>Figure S5</u>: A) Receiver Operator and B) Precision Recall curves for the validation model.

<u>Table S1:</u> Quantum Chemical Properties
<u>Table S2:</u> *E. coli* data matrix
<u>Table S3:</u> *H. sapiens* data matrix
<u>Table S4:</u> *E. coli* iRF normalized feature importance scores
<u>Table S5:</u> Frontier orbital and ground-state energies of quantum chemical properties
<u>Table S6:</u> Novel *E. coli* cutting efficiency library
